# The Relationship between Oxidative Status and Radioiodine Treatment Qualification among Papillary Thyroid Cancer Patients

**DOI:** 10.3390/cancers15092436

**Published:** 2023-04-24

**Authors:** Angelika Buczyńska, Iwona Sidorkiewicz, Maria Kościuszko, Agnieszka Adamska, Katarzyna Siewko, Janusz Dzięcioł, Piotr Szumowski, Janusz Myśliwiec, Anna Popławska-Kita, Adam Jacek Krętowski

**Affiliations:** 1 Clinical Research Centre, Medical University of Bialystok, M. Skłodowskiej 24a, 15-276 Bialystok, Poland; 2 Department of Endocrinology, Diabetology and Internal Medicine, Medical University of Bialystok, M. Skłodowskiej 24a, 15-276 Bialystok, Poland; 3 Department of Human Anatomy, Medical University of Bialystok, ul. Mickiewicza 2A, 15-230 Bialystok, Poland; 4 Nuclear Medicine, Medical University of Bialystok, M. Skłodowskiej 24a, 15-276 Bialystok, Poland

**Keywords:** papillary thyroid cancer, oxidative stress, serum, area under curve

## Abstract

**Simple Summary:**

Studies analyzing the protein profile of thyroid tissue in patients with papillary thyroid cancer (PTC) have revealed disturbed metabolic pathways, including those related to oxidative status. This study aimed to assess the concentration of specific markers associated with oxidative homeostasis in PTC patients and their potential role as screening factors for radioiodine treatment (RAI) indication and further clinical management.

**Abstract:**

Total oxidative status (TOS), total antioxidant capacity (TAC), tumor protein 53 (p53), nuclear factor kappa B (NF-κB), forkhead box protein O1 (FOXO), and sirtuin 1 (SIRT1) play crucial roles in oxidative homeostasis and the progression of papillary thyroid cancer (PTC), as previously demonstrated in the literature. Therefore, profiling these markers among PTC patients may be useful in determining their eligibility for radioiodine (RAI) treatment. Since treatment indications are based on multiple and dynamic recommendations, additional criteria for adjuvant RAI therapy are still needed. In our study, we evaluated the TOS, TAC, and serum concentrations of p53, NF-κB, FOXO, and SIRT1 to analyze the relationship between oxidative status and qualification for RAI treatment. For the purpose of this study, we enrolled 60 patients with PTC allocated for RAI treatment as the study group and 25 very low-risk PTC patients not allocated for RAI treatment as a reference group. The serum TOS and SIRT1 concentrations were significantly higher in the study group compared to the reference group (both *p* < 0.001), whereas the TAC and p53, NK-κB, and FOXO concentrations were significantly lower (all *p* < 0.05). We also demonstrated the diagnostic utility of TAC (AUC = 0.987), FOXO (AUC = 0.648), TOS (AUC = 0.664), SIRT1 (AUC = 0.709), p53 (AUC = 0.664), and NF-κB (AUC = 0.651) measurements as indications for RAI treatment based on American Thyroid Association recommendations. Our study revealed that oxidative status-related markers may become additional criteria for RAI treatment in PTC patients.

## 1. Introduction

Papillary thyroid cancer (PTC) is the most common malignant neoplasm arising from thyroid parenchymal cells [1,2]. According to the latest epidemiological data, the incidence rate of PTC is 16.1, and the mortality rate is 0.5 per 100,000 women and 0.3 per 100,000 men when adjusted for age [3,4]. In the United States, the incidence of PTC has more than tripled over the past three decades, with an annual percentage increase of approximately 4.4%. The incidence of PTC is higher in women than in men, with a female-to-male ratio of approximately 3:1. PTC is also more common in white individuals compared to other races [2,3,4].

Ultrasound-guided fine-needle aspiration biopsy (FNAB) is the gold standard for pre-operative assessment of PTC; nevertheless, up to 15% of the results are still inconsistent [5,6]. The clinical management of PTC patients is based on cancer progression, according to the American Thyroid Association (ATA) recommendations, and subsequent risk stratification [7]. A total thyroidectomy with radioactive iodine (RAI) treatment [8] is mostly recommended to eradicate potential residual disease and to improve prognosis [9,10,11,12]. However, several oxidative stress markers have been evaluated among patients who have been allocated for RAI, and significant disturbances in the oxidative status have been noted [13,14,15]. Assessing the relationship between oxidative status and PTC clinical management may reveal novel insights into RAI qualification [16]. The discrepancy between the production of free radicals and antioxidant defense has been shown to be associated with thyroid cancer. Muzza et al. demonstrated that the level of oxidative stress-related markers in PTC directly correlates with worse tumor presentation and higher tumor aggressiveness [17]. In our recent study on serum malondialdehyde concentration in PTC patients undergoing RAI, increased oxidative stress was demonstrated throughout the treatment procedure [18,19,20]. It has also been suggested that blood markers can predict early-stage RAI refractory PTC [21,22,23,24,25,26]. Our hypothesis was that there must be a relationship between oxidative stress and both the presence of PTC and distinct clinical management, including RAI.

Furthermore, tumor protein 53 (p53), nuclear factor kappa B (NF-κB), forkhead box protein O1 (FOXO), and sirtuin 1 (SIRT1) have all been shown to play important roles in regulating oxidative stress and the development of PTC [27,28,29,30,31,32]. Since many studies have demonstrated that increased oxidative stress in PTC is associated as a relevant risk factor, the level of oxidative stress-related markers in PTC has been shown to directly correlate with tumor aggressiveness [33]. Additionally, our recent study on serum malondialdehyde concentration in PTC patients undergoing radioactive iodine treatment (RAI) demonstrated increased oxidative stress throughout the treatment procedure [18]. Therefore, the measurement of parameters related to oxidative stress, including p53, NF-κB, FOXO, and SIRT1, may be useful for the clinical management of PTC patients, particularly during RAI qualification. These protein markers could be included in risk stratification criteria and used to develop personalized treatments that unify RAI recommendations.

In this study, the total oxidative status (TOS), total antioxidant capacity (TAC), and serum concentrations of p53, NF-κB, FOXO, and SIRT1 were measured to analyze the relationship between the oxidation–reduction status and qualification for RAI treatment. The study aimed to determine the potential diagnostic usefulness of these markers as indicative markers for RAI therapy.

## 2. Materials and Methods

### 2.1. Study Subjects

This research was conducted at the Department of Endocrinology, Diabetology, and Internal Medicine at the Medical University of Bialystok, Poland. All patients were diagnosed with PTC based on histopathological examinations, clinical laboratory tests, and ultrasound imaging. For this study, 60 patients who had been diagnosed with different stages of PTC, undergone total thyroidectomy, and demonstrated adjuvant indications for RAI therapy (multifocal carcinomas with angioinvasion and/or capsular infiltration, increased concentrations of thyroglobulin, Tg, and the thyroglobulin antibody, TgAb) were enrolled as the study group. Additionally, 25 PTC patients who had undergone total thyroidectomies but did not receive any recommendations for RAI therapy, qualified as very low-risk PTC patients (reference group), were also enrolled [9,34]. Therapeutic decisions in thyroid cancer were made within a multidisciplinary tumor board. The same team confirmed the pathological report in all patients to ensure consistency throughout the study. The exclusion criteria were as follows: any chronic diseases, ongoing inflammation, and additional treatment that may interfere with oxidative status.

### 2.2. Sample Collection and Measurement

Venous blood (5.5 mL) was obtained during the visit and centrifuged, which was followed by serum separation. The samples were then frozen at −80 °C.

The serum concentrations of TSH, free triiodothyronine (fT3), free thyroxine (fT4), 25-OH vitamin D (25-OH VIT D), Tg, and TgAb were measured using a Roche E411 device (Roche Diagnostics Ltd., Risch-Rotkreuz, Switzerland) via the electrochemiluminescence (ECLIA) method. The concentrations of triglyceride (TG), low-density lipoprotein (LDL), high-density lipoprotein (HDL), cholesterol (CHOL), C-reactive protein (CRP), and glucose were assayed using a Roche C111 device (Roche Diagnostics Ltd., Risch-Rotkreuz, Switzerland) via the enzymatic colorimetric method.

The TOS status was assessed using photometric immunodiagnostic assays (PerOx (TOS/TOC) Kit, KC5100, 64625 Bensheim, Germany), and the TAC status was determined using photometric assays (ImAnOx (TAS/TAC) Kit, KC5200, 64625 Bensheim, Germany). The concentrations of p53, NF-κB, FOXO, and SIRT1 were determined using enzyme-linked immunosorbent assays (ELISAs) (Enzyme-linked Immunosorbent Assay Kit, Cloud-Clone Corp., Wuhan, China; SEA928Hu, SEB824Hu, SEA764Hu and SEE912Hu, respectively), according to the manufacturer’s instructions. The samples and controls were measured in duplicate using a fully automated two-plate ELISA processing system (Dynex DS2, Chantilly, VA, USA) following the blind analysis method in the same run.

### 2.3. Statistical Analysis

Our statistical analyses were performed using the GraphPad Prism 9.0 software (GraphPad Software, Inc., San Diego, CA, USA). The preliminary statistical analysis (Shapiro–Wilk test) did not demonstrate a normal distribution for the data. Thus, nonparametric tests were used for the statistical analyses between the groups. In this paper, all the data are presented as the medians and quartiles. A Mann–Whitney *U* test for independent variables was used to examine the differences between the study and reference groups. Any correlations were determined using nonparametric Spearman’s tests. Values of *p*  <  0.05 were significant. In addition, the receiver operating characteristic (ROC) curves were determined using simultaneous sensitivity and specificity calculations.

### 2.4. Institutional Review Board Statement

The study was conducted according to the guidelines of the Declaration of Helsinki, and the procedures were approved by the Local Ethics Committee of the Medical University of Bialystok, Poland. Written informed consent was obtained from each participant (R-I-002/491/2019).

## 3. Results

### 3.1. Studied Population Characteristics

Sixty patients with incomplete resection of the thyroid gland, as confirmed by imaging tests, such as scintigraphy and/or ultrasound, were eligible for RAI based on ATA recommendations. These patients had multifocal carcinomas with angioinvasion and/or capsular infiltration, and increased concentrations of Tg and TgAb (study group).

The reference group consisted of 25 volunteers who had been diagnosed with very low-risk PTC and were not eligible for RAI treatment (Table 1).

### 3.2. Biochemical Profiling of the PTC Patients

First, the groups were compared in terms of lipid and thyroid hormone status, as well as other biochemical measurements. The concentrations of CHOL and LDL were found to be significantly higher among PTC patients who were allocated for RAI treatment, whereas the concentrations of 25-OH VIT D and HDL were lower compared to the reference group (all *p* < 0.05). Moreover, the concentrations of TG, CRP, glucose, TSH, fT3, fT4, Tg, and TgAb did not differ between the groups (all *p* > 0.05). Additionally, the TSH concentrations were suppressed in both groups due to the PTC treatments recommended (Table 2).

### 3.3. A Comparison of the Oxidative Status-Related Parameters between the Study and Reference Groups

**Figure 1:**
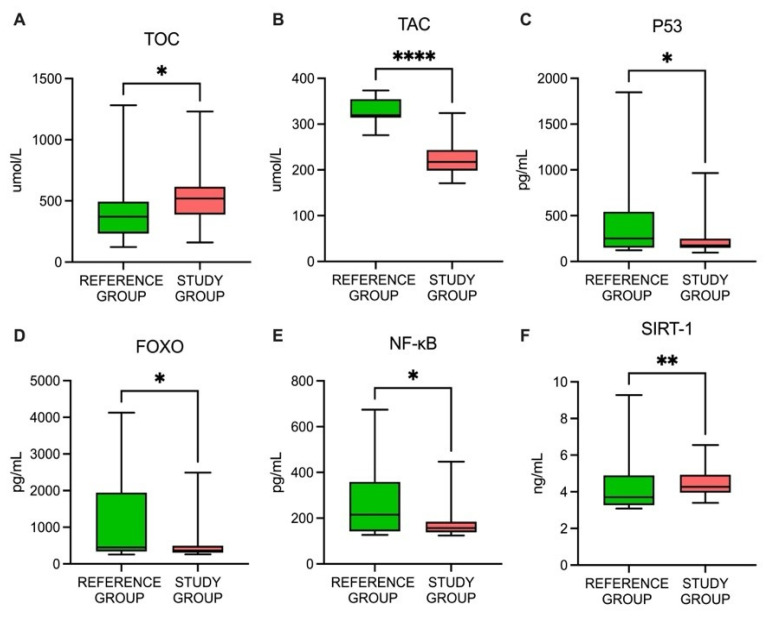
The profiles of the studied parameters: (**A**) TOC, total oxidative capacity; (**B**) TAC, total antioxidant capacity; (**C**) p53, tumor protein 53; (**D**) FOXO, forkhead box protein O1; (**E**) *NF-κB*, nuclear factor kappa B; (**F**) SIRT1, sirtuin 1; * *p* < 0.05; ** *p* < 0.01; **** *p* < 0.001.

In this study, we assessed TOC and TAC measurements as well as indirect oxidative status markers, such as p53, NF-κB, FOXO, and SIRT1, in PTC patients. The levels of oxidative status-related parameters differed significantly between the study and reference groups. The study group showed significantly increased TOC and SIRT1 concentrations compared to the reference group (*p*  <  0.05 and *p*  <  0.01, respectively). In addition, the study group demonstrated significant decreases in the TAC, p53, NF-κB, and FOXO concentrations compared to the reference group (all *p*  <  0.05) (Table 3, Figure 1).

### 3.4. The Association of the Oxidative Status-Related Parameters in PTC Patients

A Spearman regression was performed to study the relationship between the biochemical parameters. This study involved patients who had been diagnosed with different stages of PTC after thyroid resection; thus, a correlation assessment was performed on the total PTC group.

**Figure 2:**
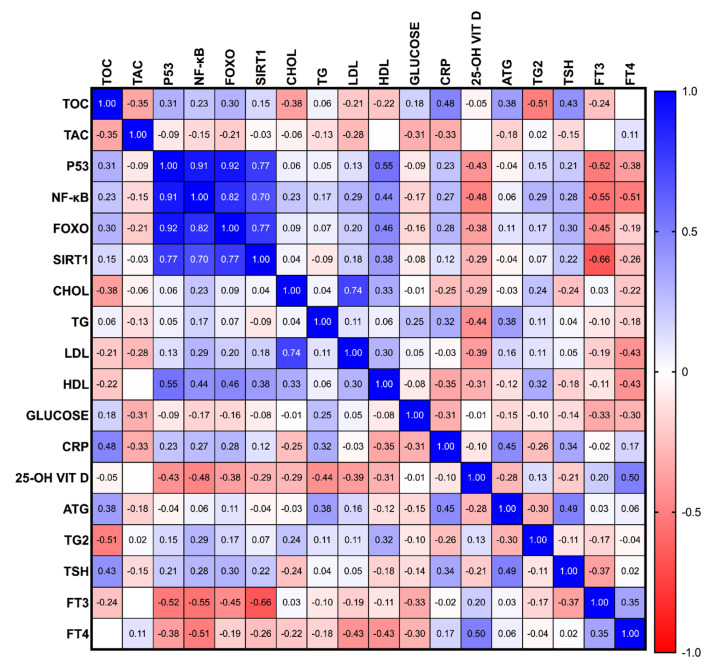
The correlation profiles of the parameters studied: FOXO, forkhead box protein O1; NF-κB, nuclear factor kappa B; p53, tumor protein 53; SIRT1, sirtuin 1; TAC, total antioxidant capacity; TOC, total oxidative capacity; CHOL, cholesterol; CRP, C-reactive protein; PTC, differentiated thyroid cancer; fT3, free triiodothyronine; fT4, free thyroxine; HDL, high-density lipoprotein; LDL, low-density lipoprotein; TG, triglyceride; TG, thyroglobulin; TGAb, thyroglobulin antibody; TSH, thyroid-stimulating hormone; 25*-OH* VIT D, 25-OH vitamin D.

Interestingly, a positive correlation was demonstrated between the p53 and FOXO concentration and between the p53 and NF-κB concentration (r = 0.92 and r = 0.91, respectively; all *p* < 0.001). Furthermore, a positive correlation was observed between FOXO and NF-κB measurements (r = 0.82; *p* < 0.01). The SIRT1 concentrations were positively correlated with the p53, NF-κB, FOXO measurements, and negatively correlated with the fT3 concentration (r = 0.77, r = 0.70, r = 0.77, r = −0.66, all *p* < 0.01). Moreover, a positive correlation between the LDL and CHOL measurements was demonstrated (r = 0.74; *p* < 0.01). In our study, a negative correlation between the TOC and Tg levels was demonstrated (r = −0.51; *p* < 0.001). A positive correlation between p53 and HDL measurements, and a negative correlation between p53 and fT3, were found (r = 0.55, r = −0.52, respectively; all *p* < 0.01). The NF-κB measurement was found to be negatively correlated with fT3 and fT4 (r = -0.55; r = −0.51, respectively; all *p* < 0.05). A positive correlation between 25-OH vitamin D and fT4 was shown (r = 0.5; *p* < 0.01) (Figure 2).

### 3.5. Diagnostic Utility of the Studied Parameters for RAI Qualification, According to Current Recommendations

**Figure 3:**
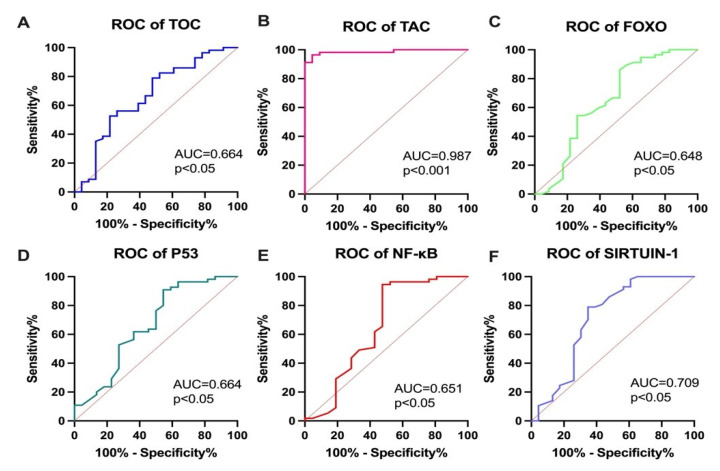
The ROC curves of the studied parameters: (**A**) TOC, total oxidative capacity; (**B**) TAC, total antioxidant capacity; (**C**) FOXO, forkhead box protein O1; (**D**); p53, tumor protein 53; (**E**) NF-κB, nuclear factor kappa B; (**F**) SIRT1, sirtuin 1.

To assess the potential diagnostic utility of the parameters selected for RAI qualification, ROC curves were constructed. The highest diagnostic utility was demonstrated for the TAC measurement (AUC = 0.987; *p* < 0.001). Some of the parameters, such as FOXO (AUC = 0.648; *p* < 0.01), TOC (AUC = 0.664; *p* < 0.01), SIRT1 (AUC = 0.709; *p* < 0.01), p53 (AUC = 0.664; *p* < 0.05), and NF-κB (AUC = 0.651; *p* < 0.05), also demonstrated screening utility (*p* < 0.05) to support the current ATA recommendations (see Figure 3).

## 4. Discussion

The FNAB procedure plays a crucial role in the preoperative assessment of PTC. The TNM classification is based on the size of the primary tumor, the presence and number of lymph node metastases, and the number of distant metastases [7,35]. The PTC risk classification system consists of multiple stages and is based on a specific combination of criteria, including the size of the primary tumor, histological examinations, angioinvasion, the infiltration of tumor cells outside of the thyroid gland, tumor metastasis, and the age at the time of diagnosis. However, the choice of management should be made together with individual patients.

Furthermore, the postoperative management of PTC patients who undergo total thyroidectomy relies on the serial measurement of serum concentrations of Tg and TgAb. However, TgAb interference limits the utility of Tg as a tumor marker in TgAb-positive patients [36,37]. Therefore, incorporating novel biochemical determinations based on oxidative stress status profiling into further patient management procedures could lead to the simplification and clarification of guidelines for describing persistent or recurrent disease in PTC patients [38,39]. Thyrocytes require several protective systems against intracellular ROS to maintain thyroid hormone synthesis, and the imbalance of oxidative status in PTC cells is known to play a crucial role in PTC development and progression [40,41]. Additionally, evaluating circulating oxidative status-related markers can be used to fully elucidate the oxidative homeostasis in cancer patients [42]. Thus, serum TOS, TAC, and the concentrations of p53, NF-κB, FOXO, and SIRT1 were evaluated to analyze the relationship between oxidative status and qualification for RAI treatment.

Young et al. suggested that PTC tissue is characterized by an imbalanced oxidative status and increased lipid peroxidation [43]. Additionally, Song et al. demonstrated that downregulation of the FOXO pathway in PTC cell lines leads to enhanced proliferation and clonogenesis, as well as decreased apoptosis [44]. Moreover, the relationship between NF-κB activation and PTC development and progression has been confirmed [29,45]. Furthermore, an increased SIRT1 concentration in PTC tissues has been observed [46]. On the other hand, decreased p53 expression has been widely described in PTC [47,48]. Our study validated the serum concentration of selected oxidative stress-related markers in PTC patients to determine their potential utility in clinical management.

The patients allocated for adjuvant RAI treatment were characterized by decreased levels of FOXO, p53, and NF-κB, and increased concentrations of SIRT1 compared to the reference group. FOXO proteins are a family of transcription factors that play important roles in the regulation of gene expression involved in cell growth, proliferation, differentiation, longevity, DNA damage, and tumorigenesis [49]. FOXO also regulates mitochondrial function and adipocyte differentiation [50]. Interestingly, FOXO also acts as a tumor suppressor in cancer, which is in agreement with our results [51]. Despite the fact that patients allocated for RAI treatment presented increased TOC levels and decreased TAC levels, FOXO concentrations were also decreased, which could potentially be associated with PTC progression [52,53]. In future studies, the relationship between RAI and FOXO concentration should be determined [53]. Based on the protective effects of this protein in many processes, targeting its activity could lead to increased bioavailability for RAI and increased antioxidant protection [54].

The role of SIRT1 in cancer, including PTC patients, has been extensively studied over the past decade. Increased thyroid tissue SIRT1 expression has been found to be associated with cancer progression and worse prognosis for PTC patients [55]. In our study, increased SIRT1 concentrations were demonstrated in PTC patients allocated to RAI, which is consistent with the results presented by other authors [56]. Moreover, SIRT1 has been shown to reduce p53-mediated apoptosis, thus, promoting tumor development and progression [57]. The most important function of activated p53 is to induce cell cycle arrest, apoptosis, and DNA repair. In a study by Marcello et al., p53 expression was found to be higher in malignant tumors compared to benign thyroid lesions, indicating that the analysis of p53 activity could be useful for PTC clinical management [58]. Recent studies have also revealed that p53 can influence mitochondrial functions by changing from a normal to an abnormal state under different stress levels [59,60]. Deregulated p53 activity is particularly unfavorable when remnant thyroid tissue is found after PTC surgery [61]. Our study revealed decreased p53 concentrations among PTC patients who were allocated for RAI, suggesting that p53 could be considered an additional factor resulting from disturbed mitochondrial functionality and decreased capacity for DNA repair [62].

In addition, it has been demonstrated that NF-κB is involved in the regulation of many genes that are involved in inflammation, cell proliferation, and apoptosis, and its overactivation has been associated with cancer cell proliferation, invasion, and survival [63,64,65,66,67,68]. Moreover, several studies have reported a correlation between NF-κB activation and resistance to chemotherapy and radiotherapy in various cancers, including PTC [69,70]. Therefore, the evaluation of NF-κB concentrations could serve as a potential biomarker for RAI therapy response and may help in the selection of patients who could benefit from this treatment. However, further studies are required to elucidate the exact role of NF-κB in PTC and its potential utility in clinical practice.

Oxidative stress is a relevant risk factor linked to thyroid cancer development and progression; therefore, we hypothesized that measuring oxidative stress levels could be useful for qualifying patients for adjuvant cancer therapy. The TAC (AUC = 0.987; *p* < 0.001) and SIRT1 (AUC = 0.709; *p* < 0.01) measurements demonstrated the highest possible diagnostic utility, suggesting their usefulness in supporting clinical management and RAI qualification of PTC patients. Additionally, prior to RAI administration, the patients who were allocated for RAI treatment were characterized by increased TOC levels and decreased TAC levels. The oxidative–antioxidant status is related to RAI qualification and could be of significant clinical relevance. Moreover, positive correlations were demonstrated between the p53 and FOXO concentrations (r = 0.92; *p* < 0.001) and between the p53 and NF-κB concentrations (r = 0.91; *p* < 0.001). Furthermore, a very strong positive correlation was noticed between FOXO and NF-κB measurements (r = 0.82; *p* < 0.01). Since p53, FOXO, and NF-κB are involved in the PI3K/Akt pathway, their dysregulation may reflect cancer progression [68]. These data suggest a complex disorder of the oxidation–reduction status between PTC patients resulting from cellular and mitochondrial origin.

Interestingly, the TOC concentrations were also negatively correlated with Tg concentrations (r = −0.51; *p* < 0.001) in PTC patients. These outcomes emphasize the relationship between thyroid function markers and oxidative status [38,69]. Since oxidative stress has been found to be a valuable PTC risk factor, and RAI therapy is linked to increased oxidative status, the studied parameters could support recommendations in the case of unclear clinical features [70]. Further exploration of potential biomarkers and therapeutic targets could provide more detailed patient stratification and personalized treatments, which could improve the clinical management of PTC [67,71].

The integration of biochemical determinations could simplify and clarify the recommended guidelines for managing PTC patients. This study presents new directions in the diagnosis and treatment of PTC, but it also has several limitations. The small size of the study groups can be considered a weakness. Although the patients did not have any chronic diseases based on their medical history and laboratory measures, they were not thoroughly screened for diseases or medications that might affect the concentration of oxidative stress markers. Additionally, the presented results are preliminary, and multicenter cohort studies are needed to confirm the hypotheses. Furthermore, the study groups should be expanded to include an RAI-refractory group or benign thyroid lesions. Nonetheless, our study identified novel possibilities for further research, particularly as it demonstrated insufficient antioxidant properties among PTC patients.

Due to the fact that radiation not only eradicates cancer cells but also can affect nearby healthy cells, it can cause side effects, especially at higher activity levels of the administered ^131^I [66]. Therefore, assessing the oxidative status in postsurgical cancer restratification could enable a more personalized selection of the ^131^I doze activity. On the other hand, the risk stratification of PTC patients is a multistage process, and, thus, novel diagnostic tools that are useful in clinical management could lead to personalized treatment regimens [67]. Additional criteria that are useful in patient selection for RAI are needed because the ATA recurrence risk stratification can be challenging to apply in real-life practice [71].

## 5. Conclusions

Oxidative stress is a significant risk factor for PTC, and RAI therapy has been shown to disrupt the oxidative balance. Therefore, evaluating the oxidative status could help guide recommendations for adjuvant RAI treatment, particularly in cases with unclear clinical features. As PTC risk stratification is a multistep process, our study highlights the potential diagnostic value of TAC and SIRT1 measurements in guiding personalized adjuvant RAI treatments. Future research should focus on long-term follow-up studies with large cohort groups to enhance the clinical management of PTC patients.

## Figures and Tables

**Table 1:** The characteristics of the study and reference groups.

	Study Group	Reference Group	*p*-Value
Number of patients	60	25	
Median age (upper and lower quartiles)	54 (51.41; 64.22)	51 (50.21; 62.58)	0.054
Sex	M: 18	M: 8	0.052
	F: 42	F: 17	0.051
Menopausal status			
Premenopausal	9	5	0.064
Postmenopausal	33	12	0.082
Stage (TNM)	pT1a(m): 11 pT1b: 15 pT1b(m): 6 pT2: 16 pT3/pT4: 12	pT1a: 25	<0.001

F, female; M, male; (m), multifocal; p, pathological; TNM, the tumor, node, and metastasis classification of differentiated and anaplastic thyroid cancer (based on the characteristics of the primary tumor site (pT)); pT1a, the size of the largest thyroid tumor ≤ 1 cm; pT1b, the size of the largest thyroid tumor > 1 cm but ≤ 2 cm; pT2, the size of the largest thyroid tumor > 2 cm but ≤ 4 cm; pT3/pT4, the size of the largest thyroid tumor > 4 cm with gross extrathyroidal extensions.

**Table 2:** The biochemical parameter profiles of the study and reference groups.

	Study Group					Reference Group				
	Unit	25% Percentile	Median	75% Percentile	Range	25% Percentile	Median	75% Percentile	Range	*p*-Value
CHOL	mg/dL	189.51	216.00	240.00	142–463	179.23	142.05	225.01	101–272	0.018
LDL	mg/dL	118.50	138.04	168.52	70–314	74.30	107.92	137.42	55.7–164.8	<0.001
TG	mg/dL	78.52	103.07	161.45	40–499	84.41	107.91	166.00	45–411	0.510
HDL	mg/dL	45.06	53.01	62.14	31–114	53.73	58.94	72.57	39.26–102.8	0.048
CRP	mg/L	15.85	22.41	29.35	7.2–82.6	1.00	1.34	2.83	0.38–4.12	0.053
GLUCOSE	mg/dL	88.07	94.14	99.53	69–248	85.12	92.22	97.15	77–117	0.297
25-OH VIT D	ng/mL	15.81	22.17	29.35	7.2–82.6	23.34	25.92	36.86	17.4–54.2	0.019
TSH	µIU/mL	0.15	0.60	2.32	0.1–68.55	0.12	0.36	0.79	0.075–1.55	0.318
fT3	pg/mL	2.21	2.56	3.01	1–6.27	2.47	2.63	2.86	1–3.27	0.754
fT4	ng/dL	0.96	1.19	1.37	0.4–2.14	1.05	1.19	1.23	0.4–1.8	0.991
Tg	ng/mL	0.51	1.10	2.53	0.04–37.05	0.04	0.09	0.29	0.04–1.13	0.078
TgAb	IU/mL	0.85	2.02	6.67	0–185	0.61	1.82	2.89	0.1–5.23	0.131

CHOL, cholesterol; CRP, C-reactive protein; PTC, differentiated thyroid cancer; fT3, free triiodothyronine; fT4, free thyroxine; HDL, high-density lipoprotein; LDL, low-density lipoprotein; TG, triglyceride; Tg, thyroglobulin; TgAb, thyroglobulin antibody; TSH, thyroid-stimulating hormone; 25-OH VIT D, 25-OH vitamin D.

**Table 3:** A comparison between the results from the study and reference groups for the studied parameters.

	Unit	Study Group				Reference Group				*p*-Value
		25% Percentile	Median	75% Percentile	Range	25% Percentile	Median	75% Percentile	Range	
TOC	Umol/L	387.5	519	615.5	161–1231	233	371	493	123–1282	0.020
TAC	Umol/L	198.7	217.4	243.4	170.8–324.3	313.9	317.2	353.3	271.4–373.3	<0.001
p53	pg/mL	149	176	249	98–966	150.5	250	543.3	123–1847	0.025
NF-κB	pg/mL	138	157	184	124–447	142	215	358	127–674	0.043
FOXO	ng/mL	0.3	0.37	0.5	0.26–2.49	0.34	0.45	1.94	0.26–4.13	0.039
SIRT1	ng/mL	3.96	4.26	4.93	3.4–6.56	3.27	3.71	4.9	3.09–9.28	0.003

FOXO, forkhead box protein O1; NF-κB, nuclear factor kappa B; p53, tumor protein 53; SIRT1, sirtuin 1; TAC, total antioxidant capacity; TOC, total oxidative capacity.

## Data Availability

The data that support the findings of this study are available from the corresponding authors, upon reasonable request.

## Abbreviations

*25-OH VIT D*	25-OH vitamin D
*AUC*	area under the ROC curve
*CHOL*	cholesterol
*CRP*	C-reactive protein
*FNAB*	fine aspiration needle biopsy
*fT3*	free triiodothyronine
*fT4*	free thyroxine
*FOXO*	forkhead box protein O1
*HDL*	high-density lipoprotein
*LDL*	low-density lipoprotein
*NF-κB*	nuclear factor kappa B
*p53*	tumor protein 53
*SIRT1*	sirtuin 1
*PTC*	papillary thyroid cancer
ROC	received operatic characteristics
*TAC*	total antioxidant capacity
*TOC*	total oxidative capacity
*TG*	triglyceride
*Tg*	thyroglobulin
*TgAb*	thyroglobulin antibody
*TSH*	thyroid-stimulating hormone
